# Immune pre-stimulation by injected yeast beta-glucan as a strategy to prevent calf diarrhea and bovine respiratory disease during the first 74 days of age

**DOI:** 10.3389/fcimb.2025.1586121

**Published:** 2025-05-27

**Authors:** Fang Yan, Zhihong Zhang, Xiaorong Zhan, Wenqian Yang, Junhu Yao, Xiurong Xu

**Affiliations:** ^1^ College of Animal Science and Technology, Northwest A&F University, Yangling, Shaanxi, China; ^2^ Animal Husbandry Division, Junlebao Dairy Co. Ltd., Shijiazhuang, Hebei, China

**Keywords:** yeast β-glucan, trained immunity, calves, diarrhea, bovine respiratory disease

## Abstract

**Introduction:**

The complexity of intestinal pathogens poses a great challenge to the prevention of infectious diarrhea in calves. Studies have shown that immune stimulants such as yeast beta-glucan can induce the innate immune system to acquire memory and improve their non-specific defense functions. This trial was conducted to evaluate the prophylactic effect of intraperitoneal injection of yeast β-glucan after birth on diarrhea in Holstein calves during the first 74 days of age.

**Methods:**

A total of 52 healthy newborn Holstein calves (body weight 39.3 ± 0.82 kg) were enrolled and randomly assigned into two groups (n = 26 in per group): 1) placebo group (CON), and 2) intraperitoneal injection with yeast β-glucan solution (0.1 g/mL, 50 mg/kg body weight) at 3 and 6 days of age (IP). The CON group received an equal volume of sterile saline at the same time. Body weight was measured monthly, and health checks and fecal consistency were evaluated daily for every calf. Jugular blood and rectal feces were collected at 7 and 30 days of age.

**Results:**

IP induced inflammation in calves, which was manifested as obvious increased levels of serum cytokines (IL-1β, IL-6, and TNF-a), immunoglobulin (IgG and IgM), and oxidative stress after 24 h, and the antimicrobial substance (defensin and secreted immunoglobulin A) in feces also significantly increased, but stimulation didn’t lead to a higher level of serum diamine oxidase (DAO). The pre-stimulation had no positive effect on growth performance or feed efficiency, but reduced the frequencies of diarrhea and bovine respiratory disease, especially during 31-60 d. Furthermore, the pre-stimulation increased the levels of serum IL-6, fecal defensin and secreted immunoglobulin A, while decreased the levels of serum DAO and malonaldehyde at 30 d. In addition, compared with the ones in the CON group, calves in the IP group showed a better rectal bacterial structure at 30 d, with a more enrichment of beneficial bacteria such as *Bifidobacterium*.

**Discussion:**

Our findings suggested that early stimulation with yeast β-glucan could be a promising strategy for reducing the frequencies of both diarrhea and BRD in calves.

## Introduction

The prevalence of diarrhea and bovine respiratory disease (BRD) was 18.9% and 11.3% in the United States of America, 29% and 39% in Canada, and 17.9% and 6.0% in Australia, respectively (Windeyer et al., 2014; Urie et al., 2018; Abuelo et al., 2019). Those two diseases accounted for 56.0% and 33.4% of the incidence of calves, respectively, and were the main diseases of calves. The number of calf deaths caused by diarrhea or BRD accounts for 32.0% and 14.1% of the total number of deaths, respectively (Urie et al., 2018).

Pathogen infections are the primary reason behind calf diarrhea, involving a diverse range of pathogens such as viruses, pathogenic bacteria, and parasites (Kolenda et al., 2015), including some newly discovered pathogens (Netea et al., 2016). This complexity makes it challenging to comprehensively vaccinate against infective diarrhea in calves. In recent years, the theory of trained immunity has been deeply studied, which brings new strategies to the prevention of unpredictable or complex infections in animals, especially young ones. Trained immunity refers to the development of immune memory by innate immune cells in response to infection by a pathogen or stimulation of its antigenic components to provide enhanced protection against later exposure to homologous or heterologous infections (Netea et al., 2020). This process is distinct from adaptive immunity, which involves the production of specific antibodies and memory T cells. The inducers of trained immunity include some pathogens (Quintin et al., 2012; Schrum et al., 2018) and their attenuated strains (Ifrim et al., 2014; Saeed et al., 2014), non-live ones (Brandi et al., 2022; Juste et al., 2022), or cell wall components (Briard et al., 2021; Brandi et al., 2022; Vuscan et al., 2024). In addition, yeast β-glucan, a cell wall component of fungi with β-1,3/1, 6- glycosidic linkages (Han et al., 2020), has also been shown to have the function of inducing immune memory in innate immune cells. Yeast β-glucan or beta-glucans from other organisms have been extensively studied as a prebiotic when added to animal feed daily (Ding et al., 2019; Pornanek and Phoemchalard, 2021; Goh et al., 2023). Reis et al (Reis et al., 2022) found that adding 2 g/d algal beta-glucan to milk replacer increased the weight of calves at 8 weeks and reduced the fecal scores during the first 28 days of age in Holstein dairy calves. As trained immunity inducers, studies have confirmed that two-time injections of yeast β-glucan induced macrophages in the blood of goats to acquire immune memory (Angulo et al., 2020). In newborn calves, in vivo β-glucan oral administration induced a trained phenotype in innate immune cells, leading to immune metabolic changes, upon ex vivo challenge with Escherichia coli (Angulo and Angulo, 2023). However, the effect of yeast β-glucan-induced trained immunity on the prevention of diarrhea and bovine respiratory disease (BRD) in dairy calves is unclear.

Therefore, this study aimed to evaluate the effects of intraperitoneal injection of yeast beta-glucan at birth on diarrhea and BRD frequency in Holstein dairy calves during lactation and early weaning. In addition, the differences of three inflammatory factors in serum and defense proteins secreted by intestinal epithelial cells in feces between the two groups after immune stimulation and combined stress were detected, to explore whether the effect of pre-stimulation on the intestinal health of calves involved the mechanism of trained immunity. Our study may provide a new strategy for improving calf health.

## Materials and methods

### Animal feeding and experimental design

**Figure 1:**
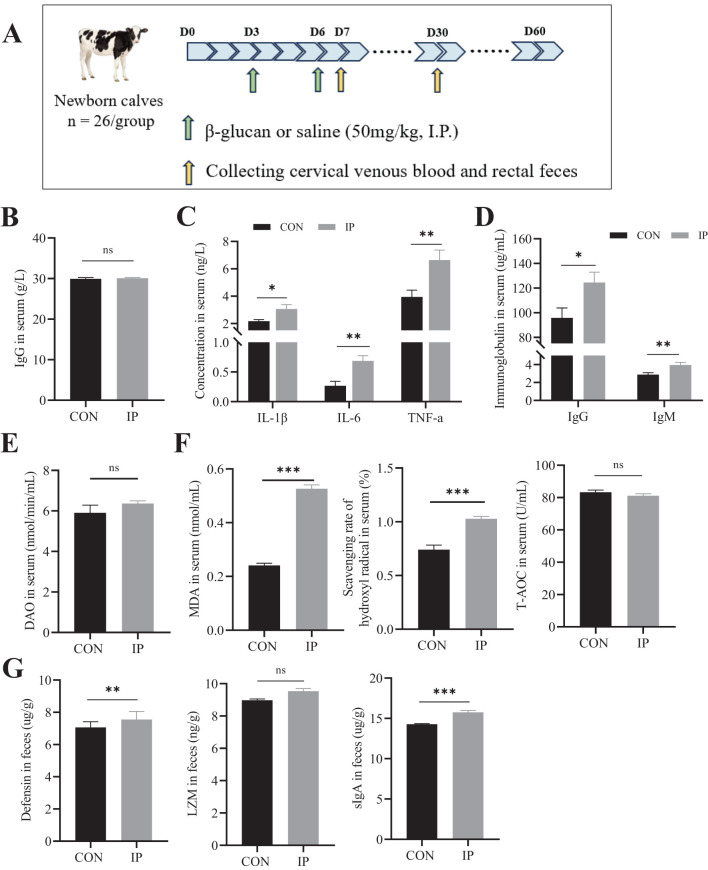
The effect of intraperitoneal injection of yeast β-glucan on calves after 24 h. **(A)** Schematic diagram of the experimental procedure. **(B)** The concentration of IgG in the serum of calves at 2 days of age. **(C)** The concentration of IL-1β, IL-6, and TNF-a in the serum of calves after intraperitoneal injection 24 h. **(D)** The immunoglobulin levels in the serum of calves after intraperitoneal injection 24 h. **(E)** The serum DAO levels were measured by ELISA to evaluate the degree of intestinal injury. **(F)** The antioxidant status of calves after intraperitoneal injection 24 h. **(G)** The Defensin, LZM, and sIgA levels in the rectal contents of calves were measured by ELISA to evaluate the degree of immune response. All data is shown as mean values ± standard error of the mean (SEM). Control group n = 12, IP group n = 15; Statistical significance was determined by the unpaired, two-tailed Student’s t-test with a 95% confidence interval, *P < 0.05, **P < 0.01, ***P < 0.001. CON, control group, IP, Intraperitoneal injection group; IL, interleukin, TNF-a, tumor necrosis factor-a; IgG, immunoglobulin G, IgM, immunoglobulin M; MDA, malonaldehyde, T-AOC, total antioxidant capacity; DAO, diamine oxidase; LZM, lysozyme; sIgA, the secreted immunoglobulin A. ns, no significance.

A total of 52 healthy Holstein heifer calves (3 d of age; BW = 39.3 ± 0.82 kg) were weighed, and housed in a naturally ventilated barn with individual shelter (3.0 m × 1.2 m × 1.8 m; length × width × height). At enrollment, the calf ID, date, and time of day (morning or evening) were recorded. Buckets with water and calf starter were available in each shelter. Screened wood shavings with a minimum theoretical length cut of 50 mm were used to minimize dustiness in the housing environment. The bedding was refreshed every 7 d to keep the pens visibly clean and dry. Calves were equally divided into two groups (n = 26 in per group) and randomly assigned to one of 2 treatments as follows: 1) placebo group (CON), and 2) intraperitoneal injection with yeast β-glucan solution (0.1 g/mL, 50 mg/kg body weight) at 3 and 6 days of age (IP). The CON group received an intraperitoneally equal volume of sterile saline at the same time. The experiment lasted until the 74th day of age. The experimental design is shown in **Figure 1A**.

Calves were fed milk 2 times daily (06:00 and 16:00) with 6 L/d, 10 L/d, and 12 L/d offered from d 2-7, d 8-20, d 21-50, respectively. At 20–23 days of age, 155 g/L commercial milk replacer with 23.4% crude protein (CP) and 13.7% fat is used to gradually replace milk according to the actual dry matter of milk. Calves were weaned over 10 d from d 51-60, receiving a total of 10 L in 2 meals/d from d 51-52, 8 L from d 53-54, 6 L from d 55-56, 4 L from d 57-58, and 2 L from d 59–60 until weaned. The composition of the milk replacer and starter concentrate is shown in **Table 1** and **Table 2**.

**Table 1:** Nutrient compositions (%, DM basis) of the milk replacer (MR).

Items	Value^1^
DM (%, as fed)	94.1
Ash	6.50
Gross energy (GE, MJ/kg of DM)	19.5
CP	23.4
EE	13.7
Ca	0.98
P	0.71

^1^Commercial milk replacer (Beijing Precision Animal Nutrition Research Center).

Items: DM, Dry matter; CP, Crude protein; EE, Ether extract.

**Table 2:** Ingredients and nutrient composition of the starter feed (%, DM basis).

Items	Value^2^
Ingredients	
Corn	36.5
Soybean meal	30.0
Wheat bran	23.4
Limestone	0.1
Premix^1^	10.0
Nutrition composition	
DM	87.63
CP	23.06
EE	3.41
CF	4.92
Ca	1.06
P	0.73

^1^Provided per kg of diet: VA 15–000 IU, VD3 5–000 IU, VE 50 mg, Fe 9 mg, Cu 12.5 mg, Mn 130 mg, Zn 100 mg, Se 0.3 mg, I 1.5 mg and Co 0.5 mg.

^2^Commercial calf starter feed (Charoen Pokphand Group agriculture and animal husbandry food enterprises).

Items: DM, Dry matter; CP, Crude protein; EE, Ether extract; CF, Crude fiber.

### Samples collection and handling

At 7 and 30 days of age, blood samples were collected from all calves by jugular venipuncture using tubes (BD Vacutainer, Franklin Lakes, NJ) at 08:00 h, subsequently centrifuged at 3000 × g for 15 min at 4°C to obtain serum. Fecal samples were collected by rectal massage in a 5 mL frozen tube at the same time. The serum and fecal samples were quickly frozen in liquid nitrogen and then kept at -40°C for subsequent analysis.

### Growth performance and health recording

To avoid the stress associated with weighing, we minimized animal weighing, and calves were weighed with mechanical scales (ICS-300; Coimma Limited) at birth, 30, and 60 days of age before the morning feeding. Milk replacer intake, Calf-starter intake, fecal consistency, and BRD score were measured daily. Feed refusals were removed before the provision of fresh starter feed. Individual feed intake was determined daily by weighing the amounts of starter feed offered and the amounts refused using a calibrated electronic scale (model PX3000; Pand Iran Co., Isfahan, Iran). Average Daily Gain (ADG) was calculated as the difference between body weight (BW) taken at 30-d intervals divided by 30. The total dry matter (DM) intake was calculated as the milk replacer DM plus the starter feed DM. The estimation of feed efficiency was calculated as the ratio of ADG to the total DM intake.

The health status of the calves was performed daily during the entire experimental period. Diarrhea was diagnosed based on fecal consistency. All calves were rectally stimulated to defecate, and fecal consistency was scored on a scale of 0 to 3, where 0 = normal consistency, 1 = semiformed or pasty, 2 = loose feces, and 3 = watery feces (Renaud et al., 2020). A fecal score ≥ 2 was considered as diarrhea. BRD and interventions were performed daily on all calves by the same trained veterinarian after the morning feeding. Signs of BRD were scored daily on each calf according to the previous study (McGuirk and Peek, 2014). Briefly, abnormal nasal discharge, coughing, ear tilt, eye discharge, and an elevated rectal temperature (TS-101 Colors Techline digital, Techline São Paulo) were recorded. The presence of at least 2 categories of abnormal scores was required for a diagnosis of BRD. The cumulative frequency of diarrhea for each group was calculated as previously described (Zou et al., 2021): The frequency of diarrhea (%) = (number of calves with diarrhea × days of diarrhea)/(total number of calves × examined days) × 100. Antimicrobial therapy was administered only when the animal showed fever or depression symptoms, such as recumbence, and decreased or refused milk intake. All calves with a positive diarrhea bout received antimicrobial intervention on the day of initial diagnosis; sulfamethoxazole and trimethoprim were administered intramuscularly with a dosage calculated by BW (1 mL/15 kg; Trissulfim, Ourofino Animal Health) according to the herd veterinarian protocol. For BRD, florfenicol + flunixin meglumine was administered intramuscularly with the dosage calculated by BW (1 mL/15 kg florfenicol; Florkem, Ceva Sante Animale; 1 mL/45 kg flunixin meglumine; Flumax, J.A. Saúde Animal) according to the herd veterinarian protocol. Medications used, dosage, and duration of treatments were recorded for individual calves.

### Detection of serum oxidative stress indicators

After removing the serum from -40°C, thaw it on ice.

The Malonaldehyde (MDA) concentration in the serum was determined using a commercially available kit (Nanjing Jiancheng Bioengineering Institute, A003-1, Nanjing, China) based on thiobarbituric acid (TBA) reactivity. Briefly, after mixing trichloroacetic acid with the homogenate and centrifuging, a supernatant was obtained, and TBA was added. The developed red color of the resulting reaction was measured at 532 nm with a spectrophotometer. Other procedures were carried out following the manufacturer’s protocols. Total antioxidant capacity (T-AOC) was detected by a colorimetry kit (Nanjing Jiancheng Bioengineering Institute, A015-1, Nanjing, China) following the manufacturer’s protocols. Hydroxy free radical scavenging activity in the serum was determined using a commercially available kit (Angle Gene Biotechnology, AK319, Nanjing, China) based on the fenton microplate process. The developed color of the resulting reaction was measured at 536 nm with a spectrophotometer.

### Detection of serum cytokines, immunoglobulins, and diamine oxidase concentrations

Cytokines (IL-1β, IL-6, and TNF-α), immunoglobulins (immunoglobulin G (IgG) and immunoglobulin M (IgM)), and diamine oxidase (DAO) in the serum were detected using a double-antibody one-step sandwich commercial ELISA kits (Jining Biotechnology, Nanjing, China) with the batch numbers JN21039, JN21752, JN20906, JN7372, and JN7340 respectively. The assays were performed using a double-antibody one-step sandwich ELISA format. The antibody was pre-coated with respective trapping antibodies sourced from commercial producer. The procedures were carried out following the manufacturer’s protocols. The absorbance (OD value) is measured at a wavelength of 450 nm using a microplate reader to calculate the sample concentration.

### Detection of intestinal antimicrobial substance concentrations

After taking the fecal samples out from a -80°C environment, thaw them on ice. After weighing, add them to the corresponding volume of sterile PBS (generally at a weight-to-volume ratio of 1:9, for example, 1 g of rectal feces sample corresponds to 9 mL of PBS) in a sterile centrifuge tube, and grind thoroughly on ice. Finally, centrifuge the homogenate at 5000×g for 5 to 10 minutes, and collect the supernatant for detection.

Defensin, LZM, and secreted immunoglobulin A (sIgA) in the rectal feces were detected using commercial ELISA kits (Jining Biotechnology, Nanjing, China) with the batch numbers JN1350, JN2132, and JN2203 respectively according to the manufacturer’s instructions.

### 16S rRNA gene sequencing of intestinal bacteria

Fifteen healthy calves that had not been treated with antibiotics before 30 days of age were randomly selected in each group for detecting the bacterial structure in the rectal feces of calves on 30 d. Bacterial genomic DNA from rectal feces samples was extracted using the CTAB according to the manufacturer’s instructions. The barcoded PCR primers F341 (5′-CCTAYGGGRBGCASCAG-3′) and R806 (5′-GGAC TACNNGGGTATCTAAT-3′) were used to amplify the V3-V4 region of the 16S rRNA gene. PCR amplification was performed in a total volume of 25 μL reaction mixture containing 25 ng of template DNA, 12.5 μL PCR Premix, 2.5 μL of each primer, and PCR-grade water to adjust the volume. The PCR conditions to amplify the prokaryotic 16S fragments consisted of an initial denaturation at 98°C for 30 s; 32 cycles of denaturation at 98°C for 10 s, annealing at 54°C for 30 s, and extension at 72°C for 45 s; and then final extension at 72°C for 10 min. The PCR products were detected with 2% agarose gel electrophoresis. Throughout the DNA extraction process, ultrapure water, instead of a sample solution, was used to exclude the possibility of false-positive PCR results as a negative control. The PCR products were purified by AMPure XT beads (Beckman Coulter Genomics, Danvers, MA, USA) and quantified by Qubit (Invitrogen, USA). The amplicon pools were prepared for sequencing and the size and quantity of the amplicon library were assessed on Agilent 2100 Bioanalyzer (Agilent, USA) and with the Library Quantification Kit for Illumina (Kapa Biosciences, Woburn, MA, USA), respectively. The libraries were sequenced on the NovaSeq PE250 platform according to the manufacturer’s recommendations, provided by LC-Bio. Alpha diversity and beta diversity were calculated by normalizing to the same sequences randomly. For alpha diversity, Chao1 and Shannon’s indices were used to evaluate the differences in microbial richness and diversity, and PCA based on unweighted UniFrac distances and PCoA based on bray-Curtis metrics were to examine the community structures of the feces microbiotas. The graphs were computed using normalized data in R (version 4.1.2) with the vegan package. To compare the abundances of the microbiome between the CON group and IP group, a Linear Discriminant Analysis Effect Size (LEfSe) was performed with LDA > 3 (*P* < 0.05) as the critical value. These data are available in NCBI with PRJNA1155013.

### Statistical analyses

Data were managed in Excel (Microsoft) spreadsheets. All statistical analyses were performed considering the calf as the experimental unit and using SPSS Statistics V21.0. The experimental design used was a randomized block, considering birth date, and birth weight as blocking factors. Baseline measurements were compared using one-way ANOVA after confirming normality (Shapiro-Wilk test *P* > 0.05 for all variables) and homogeneity of variances (Levene’s test). No significant differences were observed between groups for any baseline parameter (all ANOVA *P* > 0.05). Measurements for serum analytes, rectal temperature, and respiration frequency at all time points were analyzed for normality using a Shapiro-Wilk test, with Q-Q plots visually confirming distributions, for homogeneity of the variances using the Levene test. The treatment means were compared using the Tukey-Kramer adjustment test. The threshold of significance was set at *P* < 0.05. The figures for data visualization were performed using GraphPad Prism 9.0 (GraphPad Software Inc., San Diego, CA). The data were presented as mean ± SEM. A *P* < 0.05 was considered as statistically significant, and *P-*values between 0.05 and 0.10 represent a statistical trend. The asterisks indicate statistical significance (∗, *P* < 0.05; ∗∗, *P* < 0.01; ∗∗∗, *P* < 0.001).

## Result

### Animal inclusion/exclusion criteria

Inclusion Criteria: A total of 52 Holstein heifer calves (3 d of age; body weighing 38–40 kg at enrollment. Clinically healthy (no diarrhea/respiratory) with normal rectal temperature (<39.5°C) and success-of-passive-transfer (IgG >10 g/L). Exclusion Criteria: Animals with congenital defects, or fecal score >2 at baseline. No animals died during the experiment, and all animals were included in the statistics of diarrhea and BRD incidence frequency (n = 52). However, animal individuals with more than 2 cases illness requiring antibiotics were excluded and the final sample size during laboratory testing was: control group: n = 12 (original n= 26–14 exclusions). IP Group: n= 15 (original n = 26–11 exclusions). Excluded animals are not included in statistical analyses.

### Transient inflammatory response

We first measured the serum IgG levels of all the calf subjects at the time of their inclusion in the trial. The results are shown in **Figure 1B**, and there was no significant difference in the initial immune status between calves in the IP and CON group (*P* > 0.05). The short-term effects (24 hours after the second injection) of IP are shown in **Figures 1C-F**. In the IP group, the cytokines IL-1β (*P* = 0.019), IL-6 (*P* = 0.001), and TNF-a (*P* = 0.005), and the IgG (*P* = 0.020) and IgM (*P* = 0.007) were significantly increased. Additionally, serum MDA content and hydroxyl free radical scavenging ability were also significantly elevated (*P* < 0.001) (**Figure 1F**), but there was no significant difference in DAO content between the two groups (*P* = 0.263) (**Figure 1E**). In the rectal contents, the levels of antibacterial substances secreted by intestinal epithelial cells, namely defensin (*P* = 0.005) and sIgA (*P* < 0.001), were significantly increased, while there was no significant difference in LZM content (*P* > 0.05).

### Frequency of diarrhea and BRD

**Figure 2:**
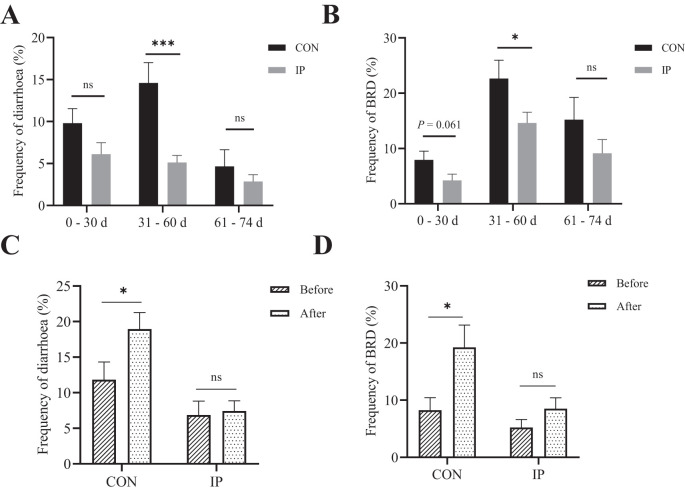
Pre-stimulation with yeast β-glucans decreased the frequency of diarrhea and BRD in calves. **(A)** The frequency of diarrhea and **(B)** BRD during 0–74 days of age. **(C)** The frequency of diarrhea and **(D)** BRD 2 weeks before and after 30 days of calves. All data is shown as mean values ± standard error (SEM). n = 26 in per group; Statistical significance was determined by the Chi-squared test with a 95% confidence interval, *P < 0.05, ***P < 0.001. CON, control group, IP, intraperitoneal injection group, BRD, Bovine Respiratory Disease. ns, no significance.

As presented in **Figures 2A, B**, the overall frequency of diarrhea and BRD were both reduced by IP, especially from 31–60 days period the frequencies of both diseases were observed significantly lower (*P* < 0.001). Moreover, the frequency of diarrhea (*P* = 0.041) and BRD (*P* = 0.019) in the CON group of calves significantly increased when comparing the 2 weeks before and after reaching 30 days of age. Whereas in the IP group, the frequency of both diseases remained stable with no significant increase during the same period (*P* > 0.05) (**Figures 2C, D**).

### Growth performance

As outlined in **Table 3**, there were no significant differences in the intake (milk replacer, calf-starter feed, and total DM), ADG, and feed efficiency between the CON group and the IP group throughout the entire 0–60 d period (*P* > 0.05).

**Table 3:** Effects of prestimulation with yeast β-glucans on the growth performance of calves.

Items	Experimental groups		SEM	*P*-value
	CON	IP		
0-30d				
Milk replacer intake, L/d	8.509	8.431	0.086	0.372
Calf-starter intake, kg/d	0.035	0.039	0.005	0.680
Total DM intake*, kg/d	1.272	1.263	0.015	0.566
ADG(kg)	0.945	0.954	0.031	0.879
Feed efficiency^1^	0.744	0.756	0.051	0.801
31-60d				
Milk replacer intake, L/d	9.356	9.562	0.117	0.086
Calf-starter intake, kg/d	0.120	0.085	0.009	0.056
Total DM intake*, kg/d	1.467	1.464	0.028	0.918
ADG(kg)	1.037	1.031	0.030	0.917
Feed efficiency	0.698	0.703	0.048	0.934
0-60d				
Milk replacer intake, L/d	8.945	8.997	0.081	0.524
Calf-starter intake, kg/d	0.078	0.060	0.012	0.154
Total DM intake*, kg/d	1.366	1.356	0.019	0.609
ADG(kg)	0.991	1.002	0.015	0.465
Feed efficiency	0.723	0.733	0.013	0.445

*Total DM intake = milk replacer DM + starter feed DM.

^1^Feed efficiency was calculated by dividing ADG by average total DMI (milk replacer DM + starter feed DM).

Items: DM, Dry matter; ADG, Average Daily Gain. Experimental groups: CON, Control group; IP, Intraperitoneal injection group.

### Oxidative stress status

**Figure 3:**
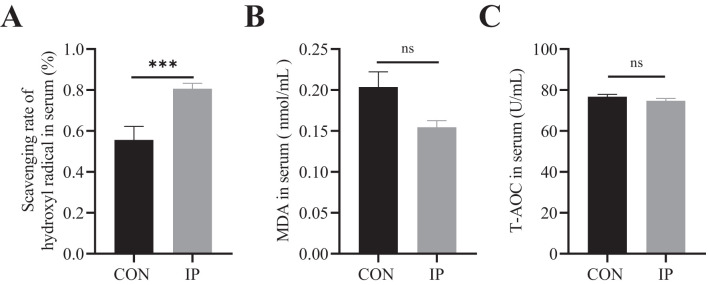
The effect of pre-stimulation with yeast β-glucans on oxidative stress level of calves. **(A-C)** The antioxidant status in the serum of calves. All data is shown as mean values ± standard error (SEM). Control group n = 12, IP group n = 15; Statistical significance was determined by the unpaired, two-tailed Student’s t-test with a 95% confidence interval or One-way ANOVA analysis, ***P < 0.001. CON, control group, IP, intraperitoneal injection group. MDA, Malonaldehyde, T-AOC, total antioxidant capacity. ns, no significance.

The difference in oxidative stress levels between the two groups at 30 days of age was shown in **Figures 3A-C**. The hydroxyl radical scavenging ability of calves in the IP group was significantly higher than that in the CON group (*P* < 0.001) (**Figure 3A**), while the level of MDA in the IP group was lower than CON group (*P* = 0.076) (**Figure 3B**), but there was no obvious difference in serum total antioxidant capacity (T-AOC) between the two groups (*P* > 0.05) (**Figure 3C**).

### Concentrations of serum cytokines, immunoglobulins, diamine oxidase, and defensive proteins in rectal contents

**Figure 4:**
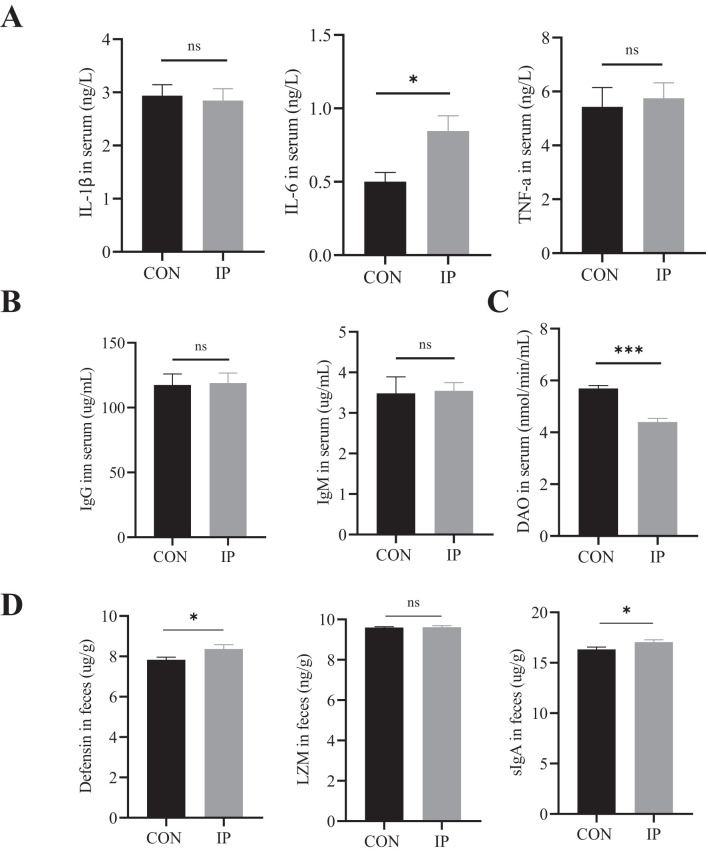
The effect of pre-stimulation with yeast β-glucans on the cytokines, immunoglobulins, DAO in serum, and intestinal antimicrobial substance Levels in rectal contents. **(A)** The concentrations of IL-1β, IL-6, and TNF-a in the serum were measured by ELISA to evaluate the degree of immune response. **(B)** The immunoglobulin levels and **(C)** DAO levels in the serum of calves were measured by ELISA to evaluate the degree of immune response and intestinal injury. **(D)** The Defensin, LZM, and sIgA levels in the rectal contents of calves were measured by ELISA to evaluate the degree of immune response. All data is shown as mean values ± standard error (SEM). Control group n = 12, IP group n = 15; Statistical significance was determined by the unpaired, two-tailed student’s t-test with a 95% confidence interval or One-way ANOVA analysis, *P < 0.05, ***P < 0.001. CON, control group, IP, intraperitoneal injection group. TNF-a, Tumor necrosis factor-a; IL, Interleukin; DAO, diamine oxidase; LZM, lysozyme; sIgA, the secreted immunoglobulin A. ns, no significance.

At 30 days of age, the serum IL-6 level in the IP group was significantly higher than that in the CON group (*P* < 0.001), while the serum DAO content was significantly lower (*P* = 0.023) (**Figures 4A, C**). There were no differences in serum levels of IL-1β, TNF-α, IgG, and IgM between the two groups (**Figures 4A, B**), but in the rectal contents, the levels of defensin (*P* = 0.047) and sIgA (*P* = 0.043) were significantly higher than that in the CON group.

### Rectal bacterial communities

**Figure 5:**
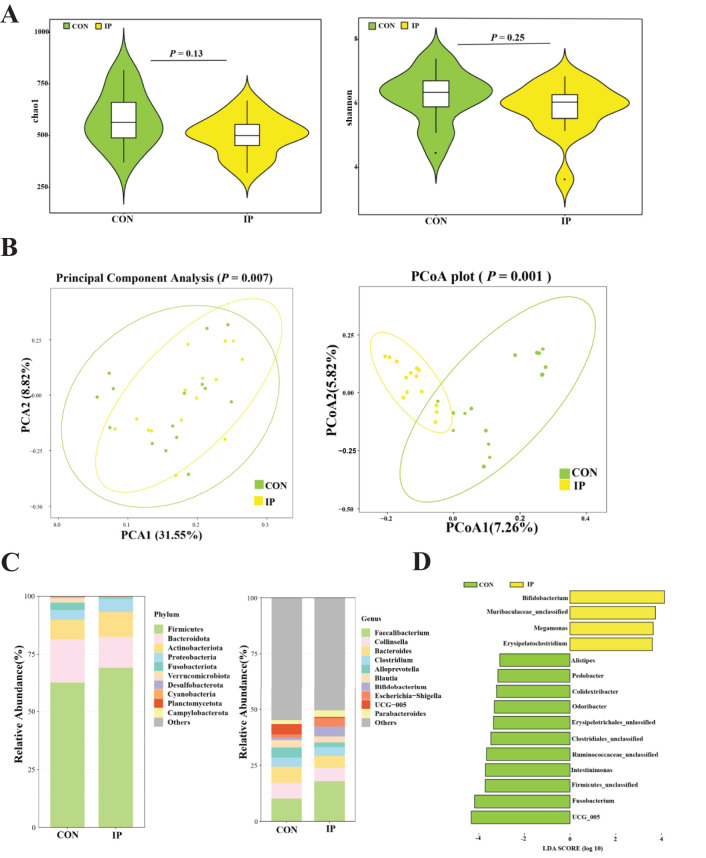
The effect of pre-stimulation with yeast β-glucans on the rectal bacterial community of calves. **(A)** Chao1 and Shannon index on the ASVs level. **(B)** Principal Component Analysis (PCA) and Principal coordinate analysis (PCoA) based on bray-curtis. **(C)** Microbial composition at the phylum level and genus level. **(D)** Linear discriminant analysis effect size of fecal bacterial microbiota (LDA > 3, *P* < 0.05).

As shown in **Figure 5A**, there was no significant difference in the richness (*P* = 0.13) and diversity (*P* = 0.25). However, as presented in **Figure 5B**, the IP group was separated from the CON group (*P* < 0.01), and the individuals of the IP group clustered together, while those of the CON group scattered.

The relative abundance and composition of the top 10 abundance at the phylum level are shown in **Figure 5C**. The rectal bacterial community in calves was dominated by *Firmicutes* and *Bacteroidota*, followed by *Actinobacteriota*. At the genus level, *Faecalibacterium* was the main genera, followed by *Collinsella*, *Bacteroides*, *Clostridium*, *Alloprevotella*, *Blautia*, *Bifidobacterium*, *Escherichia-Shigella*, *UCG-005*, and *Parabacteroides*. As shown in **Figure 5D**, it was found that *Bifidobacterium*, *megamonas*, and *Erysipelatoclostridium* were significantly enriched in the IP group (*P* < 0.05), while *Alistipes*, *Pedobacter*, *Colidextribacter*, *Odoribacter*, *Intestinimonas*, *Fusobacterium*, and *UCG-005* in the CON group were significantly more abundant than those in the IP group (*P* < 0.05).

### Functional prediction

**Figure 6:**
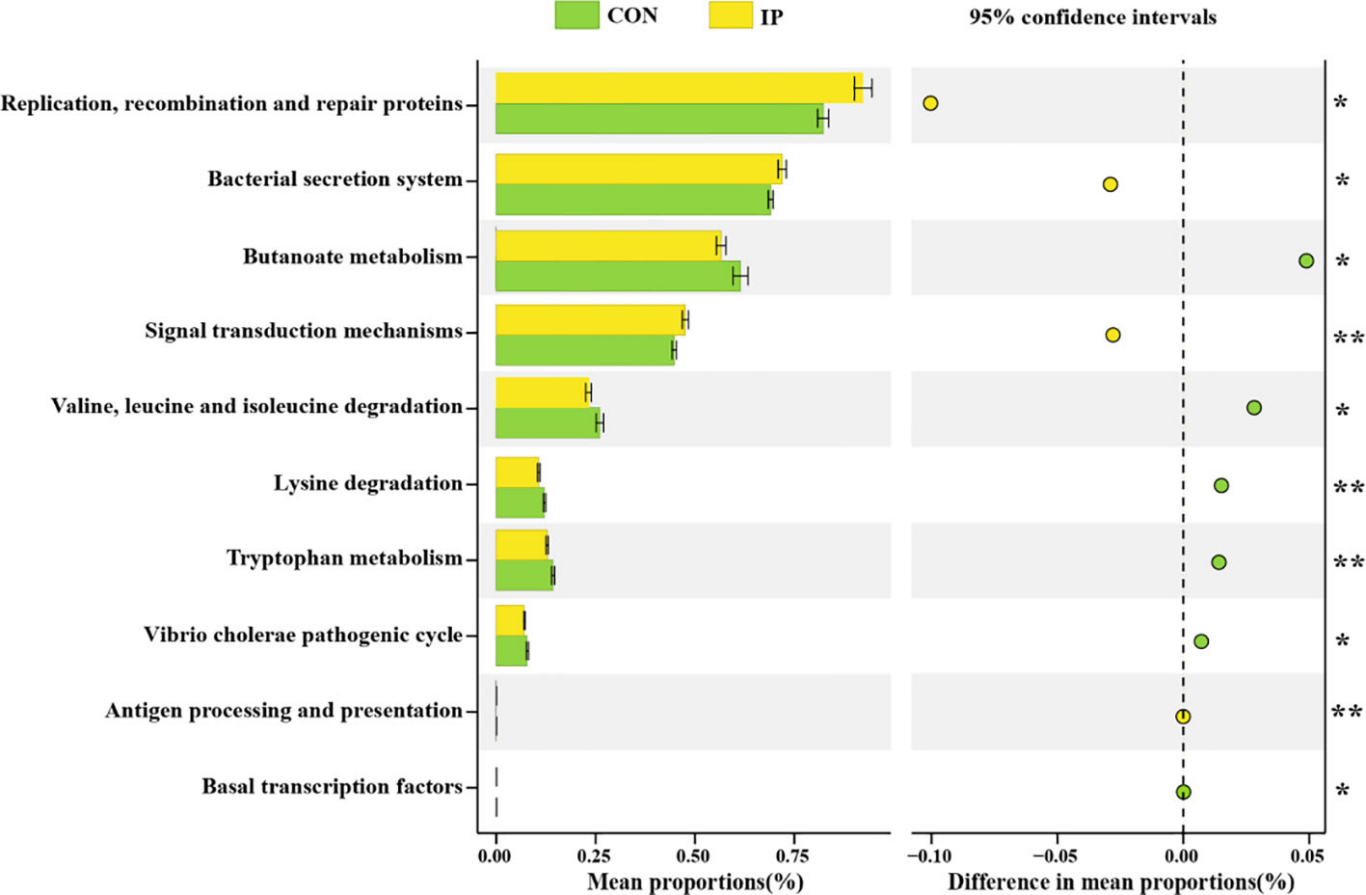
The functional prediction of rectal bacterial community of calves. Differential species T-test analysis of rectal bacterial community of calves at the phylum level. Statistical significance was determined by the unpaired, two-tailed student’s t-test with a 95% confidence interval or One-way ANOVA analysis. *P < 0.05, **P < 0.01.

The results in **Figure 6** showed that at level 3 of the Kyoto Encyclopedia of Genes and Genomes (KEGG) pathway, bacterial functional characteristics were mainly enriched in immune pathways, such as signal transduction mechanisms, bacterial secretion system, replication, recombination, and repair proteins. The differences in metabolic pathways of functional genes in bacterial flora between the two groups showed that the two functional pathways of butyrate metabolism (*P* < 0.05) and tryptophan metabolism (*P* < 0.01) in the IP group were significantly weakened. At the same time, amino acid degradation pathways, including valine, leucine, and isoleucine degradation (*P* < 0.05), and lysine degradation (*P* < 0.01) in the IP group were less active.

## Discussion

The suckling period is critical for calves. The immune system of animals at this period is undeveloped, therefore, animals are very susceptible to infection at this stage. Pathogenic infection often leads to diarrhea and BRD in calves despite the use of several vaccines and antibiotic alternatives (Vlasova and Saif, 2021). These two diseases are the most common ones in calves and are the leading causes of mortality (Abuelo et al., 2019). Moreover, severe diarrhea or pneumonia, even if well treated, may affect a calf’s later growth performance and milk production in adulthood (Abuelo et al., 2019). Therefore, research on the effective prevention of intestinal or respiratory infections in calves is urgent for both dairy and beef cattle farming. In this study, we investigated the effect of twice intraperitoneal injection of yeast β-glucan during the first week of birth on the prevention of diarrhea and BRD of dairy calves and proved that the treatment decreased both the frequency of diarrhea and BRD during the first 74 days of age.

Yeast β-glucan is generally considered both a prebiotic and immune-modulator. Several studies have shown the positive effects of oral supplementation on calf health and growth performance as prebiotics (Ma et al., 2015; Xiao et al., 2016);. Based on the theory of trained immunity of fungal β-glucan (Kalafati et al., 2020), we hypothesized that intraperitoneal injection of it early in life might induce the innate immune system to produce immune memory and effectively prevent intestinal infections, thereby reducing the frequency of diarrhea and even BRD during the early life of calves. As expected, yeast beta-glucan pre-stimulation had a positive effect, significantly reducing the frequency of diarrhea between 31 and 60 days of age. From the detected intestinal damage marker, it can be inferred that pre-stimulation with yeast β-glucan showed a systemic prophylactic effect, which improved the intestinal health status of calves later.

The systemic preventive mechanism might be complex but should include the immune memory acquired by the body’s innate immune cells due to the pre-stimulation. Studies in experimental mice or rats have shown that pre-stimulation with special antigens enables bone marrow stem cells and progenitor cells to acquire immune memory for several months to a year (for the PROMISE- EBF Study Group et al., 2015), and the acquired memory can be passed on to differentiated bone marrow cells including innate immune cells in the blood and resident ones in tissues (Mitroulis et al., 2018; Wang et al., 2023), such as ILC3 in intestine (Serafini et al., 2022) and alveolar macrophages in lung (Jeyanathan et al., 2022), and thus improve the ability of various tissues to fight later infections. Guerra-Maupome et al (Guerra-Maupome et al., 2019) reported that *in vivo* stimulation with Bacille Calmette-Guerin induced a trained innate immune phenotype in calves, characterized by increased levels of several inflammatory factors upon re-stimulation. In the present study, the levels of serum IL-6 and rectal defensin secreted by intestinal epithelial cells in pre-stimulated calves were significantly higher than those in the CON group at 30 days of age, suggesting that pre-stimulation might induce related cells to acquire immune memory in calves.

The classical study of trained immunity consists of three stages, namely pre-stimulation, resting period, and re-stimulation (Netea et al., 2020). In feeding experiments, it is difficult to determine when infection occurs after pre-stimulation in each calf. However, in our experiment, all of the investigated calves underwent a series of stresses before and after 30 days of age, including milk replacement, abrupt drop in temperature, and taking off the waistcoat. The frequency of diarrhea and BRD in the CON group increased significantly two weeks after 30 days of age, indicating that this series of stresses worsened the intestinal and respiratory health of the calves. Stresses been proven to be associated with an increased risk of infection (Nabenishi and Yamazaki, 2017; Qiao et al., 2023), and thus the combined stress could be considered as the re-stimulation phase of the classical trained immunity studies. Elevated pro-inflammatory factors generally indicate an inflammatory response, but are also often a signal of a positively enhanced defense response of innate immune cells, as proven by trained immunity studies, where increased gene expression and release of cytokines, such as TNF-α, IL-1β, and IL-6 indicates a more effective defense response (Arts et al., 2018). In this study, the lower frequency of diarrhea and BRD and higher serum IL-6, defensin, and sIgA levels in the pre-stimulation group suggested that pre-stimulation increased the immune response of calves to above stresses, and thus improved their intestinal and respiratory health. Previous studies detected that *in vitro* training with yeast β-glucan enhanced the production of TNF-α and IL-6 in macrophages and monocytes upon secondary stimulation (Angulo et al., 2020; Paris et al., 2020). Similarly, an *in vivo* study showed up-regulation of gene transcription and higher production levels of TNF-a and IL-6 in mice trained with β-glucan upon an ex vivo challenge with *E. coli* (Geervliet et al., 2020). Although the fact that the expression of TNF-α at 30 days of life was unaffected by our treatment seemed to make it complicated to explain, there is evidence that the inflammatory factors that are elevated in response to later re-stimulation vary from one trained immune study to another, due to different stimulants, different cell types, different hosts, or different re-challenge studied (Mitroulis et al., 2018; Jeyanathan et al., 2022).

Pieces of evidence proved that oral administration of yeast β-glucan daily results in an improved gut microbial balance. For instance, Zhou et al. (Horneck Johnston et al., 2024) observed the counts of pathogenic *E. coli* decreased and the counts of commensal Lactobacillus increased in yeast β-glucan supplemented calves compared with controls, and Virginio Junior et al (Virginio Junior et al., 2021) reported that the administration of β-glucan daily increased the abundance of *Alloprevotella*, a genus associated with improved intestinal barriers and tighter epithelial junctions in the lower gut. Our study also showed that pre-stimulation with yeast β-glucan optimized the bacterial structure in the rectal content of calves, including enhancing the abundance of some beneficial bacteria and stabilizing the bacterial community, as the intestinal bacterial structure tended to be consistent among individual calves in the pre-stimulated group. However, the addition of yeast β-glucan as an immune stimulant and prebiotics has different mechanisms of influence on the intestinal microflora. The former affects the intestinal microflora mainly by regulating the immune response of the intestine and even the body (Novak and Vetvicka, 2008; Quintin et al., 2012), while the latter’s main function is to promote the proliferation of beneficial bacteria as a fermentation substrate, although some studies have reported that it is also involved in regulating the intestinal mucosal immunity (Samuelsen et al., 2014; So et al., 2021). Despite the improved intestinal health status, the present study found that ADG and feed efficiency of calves were not improved by the pre-stimulation with yeast β-glucan. However, positive effects of oral administration with β-glucan daily on ADG and feed efficiency were found (Abo Ghanima et al., 2020; Reis et al., 2022). We speculated that the reasons for the different effects of the two supply modes of beta-glucan on growth performance may involve the following aspects: firstly, as an immune stimulant, the total amount of β-glucan consumed during the present trial is far lower than the total amount as a daily supplement of prebiotics; Secondly, compared with the CON group, the obvious immune response of the pre-stimulated calves was induced during the stimulation period, and the immune level of the calves was also higher when they were affected by the combined stress at about 30 days of age. This meant that during both phases, the pre-stimulated calves might expend more energy for the immune response, but the daily feed intake does not increase, and this probably resulted in no increase in daily gain despite the improved health of the pre-stimulated calf. Nevertheless, it is necessary to compare the effects of two yeast β-glucan supply modes on the prevention of diarrhea and BRD in calves in the same feeding trial, as well as subsequent effects on calves, including their adult performance.

Pre-stimulation through intraperitoneal injection will induce inflammatory response. As we observed, the level of the three detected inflammatory factors in serum increased 24 h after the stimulation with yeast β-glucan. From the theory of trained immunity and its research reports, the inflammatory response caused by pre-stimulation is a condition for the relevant cells to acquire trained immunity (Netea et al., 2020; Ochando et al., 2023). Studies in mice and rats showed that the induced inflammatory response is generally transitory and disappears within two or three days (Cheng et al., 2014; Horneck Johnston et al., 2024). It can be seen that the stimulation did not cause the increase of DAO in serum, and it was speculated that the inflammatory response induced by pre-stimulation did not cause damage to the intestinal tissue of calves in the present study. However, the sharply increased MDA level in serum indicated that the oxidative stress level of the body increased due to the stimulation. Of course, the level of serum oxidative stress in our pre-stimulated calves tended to be lower than that in control calves at 30 days of age, indicating better physical health of pre-stimulated calves later. Nevertheless, whether the transitory negative effects of pre-stimulation on calves are related to the absence of positive effects on growth performance, and whether reducing the injected dose of yeast beta-glucan can achieve the same defensive effect while eliminating oxidative stress in calves, remains to be explored.

In summary, the present study showed that intraperitoneal injection of yeast-derived β-glucan early in life effectively reduced the frequency of diarrhea and BRD from d 31 to 60, improved the intestinal health status of suckling Holstein dairy calves and suggested the involvement of trained immunity. However, more works are needed to investigate whether pre-stimulation with yeast β-glucan or other stimulants has long-term health benefits or future product performance of calves. In addition, the cytokine changes caused by training immunity may not only be manifested in IL-1β, IL-6, and TNF-a, but also may include IL-8, IL-10, and IFN, etc. The selection of markers for training immunity needs further optimization. Furthermore, the better supplementation method, time, and dosage of stimulants, etc. all need further studies.

## Data Availability

\The datasets presented in this study can be found in online repositories. The names of the repository/repositories and accession number(s) can be found below: https://www.ncbi.nlm.nih.gov/, PRJNA1155013.
